# The impact of social health insurance on rural populations

**DOI:** 10.1007/s10198-021-01268-2

**Published:** 2021-02-27

**Authors:** Colin Green, Bruce Hollingsworth, Miaoqing Yang

**Affiliations:** 1 Present Address: Department of Economics, Norweigian University of Science and Technology, Trondheim, Norway; 2 Division of Health Research, Lancaster University, Lancaster, LA1 4YX UK; 3 Department of Economics, Lancaster University Management School, Lancaster University, Lancaster, LA2 0PF UK; 4 Present Address: National Perinatal Epidemiology Unit, Nuffield Department of Population Health, University of Oxford, Oxford, OX3 7LF UK

**Keywords:** Social health insurance, Medical care utilisation, Health facility choice, Out-of-pocket payments, China, D04, I13, I18

## Abstract

Improving health outcomes of rural populations in low- and middle-income countries represents a significant challenge. A key part of this is ensuring access to health services and protecting households from financial risk caused by unaffordable medical care. In 2003, China introduced a heavily subsidised voluntary social health insurance programme that aimed to provide 800 million rural residents with access to health services and curb medical impoverishment. This paper provides new evidence on the impact of the scheme on health care utilisation and medical expenditure. Given the voluntary nature of the insurance enrolment, we exploit the uneven roll-out of the programme across rural counties as a natural experiment to explore causal inference. We find little effect of the insurance on the use of formal medical care and out-of-pocket health payments. However, there is evidence that it directed people away from informal health care towards village clinics, especially among patients with lower income. The insurance has also led to a reduction in the use of city hospitals among the rich. The shift to village clinics from informal care and higher-level hospitals suggests that the NRCMS has the potential to improve efficiency within the health care system and help patients to obtain less costly primary care. However, the poor quality of primary care and insufficient insurance coverage for outpatient services remains a concern.

**Supplementary Information:**

The online version contains supplementary material available at 10.1007/s10198-021-01268-2.

## Introduction

Financial constraints are one of the major causes of insufficient health care and impoverishment for poor people in developing countries [1]. A lack of financial protection prevents people from accessing necessary health care and puts them at risk of financial hardship following payments for health services [2]. To tackle these issues, many low- and middle-income countries have introduced state-sponsored insurance programmes for people working in informal sectors with the aim of enhancing access to healthcare and providing financial protection from the burden of illness [3]. In China, a social health insurance programme, the New Rural Cooperative Medical Scheme (NRCMS), was introduced in 2003. It was targeted at rural areas, where 80% of people (about 640 million) were not covered by any kind of health insurance following the market liberalisation reforms of the late 1970s [4]. Large government subsidies were provided for those who are enrolled in the NRCMS and particularly protect the poor against financial impoverishment caused by unaffordable care. The programme was implemented on a county-by-county basis from 2003, and by the end of 2008, it covered nearly all rural counties.

As might be expected with a programme of such scale, there has been interest in evaluating the impact of the NRCMS on health care utilisation, medical expenditure and health outcomes. Findings have been mixed. Some have shown that the NRCMS increased outpatient and inpatient use of health services [5–7], while others have found little evidence that having insurance increased overall utilisation [8–11]. There appears to be an increase in the likelihood of seeking care from primary care facilities after insurance was introduced [5, 6, 8, 10–13]; however, the quality of care delivered by primary care facilities is found to be poor [14–16]. In addition, the NRCMS seems to provide a limited degree of financial protection towards insured households, and the heavy financial burden of health care remains a challenge [6, 12, 13, 17, 18]. Most previous literature focused on the first few years of the policy implementation or in some cases only examined a handful of pilot regions, so there is a lack of recent and comprehensive evidence on the impact of the NRCMS. In addition, little evidence exists on the quality of care received by insured patients. Given that the NRCMS reimbursement policies aim to promote initial contact with primary care facilities, questions remain whether these grass-root facilities are able to perform a gate-keeping function and provide good quality services. If the quality of care remains to be low, the NRCMS will not lead to actual gains in population health even with better access to health care [14].

Following the inception of the insurance in 2003, this paper aims to explore its long-term impact using the latest waves of data (2009, 2011 and 2015) from a large national panel dataset, the China Health and Nutrition Survey (CHNS). It is a longitudinal survey with ten waves since 1989 and includes a wide range of demographic and socio-economic characteristics as well as measures of perceived health care access and expenditures. Our paper contributes to the existing literature in a number of ways. First, we not only examine the effect of coverage on overall health care utilisation, but also explore more specifically patients’ treatment-seeking responses in terms of their choices of health providers. This is important as the reimbursement rules and quality of care vary significantly across levels of facilities and types of care. Hence, the findings are informative in terms of whether the NRCMS helps patients obtain good quality services likely to lead to actual gains in population health. Second, we employ an instrumental variable (IV) estimation approach to account for the unobserved confounding factors by exploiting the staggered implementation of the NRCMS across counties over time. To strengthen the validity of the IV, we control for differences in the urbanicity level of the villages as well as year and province fixed effects. In this case, the roll-out of the NRCMS across counties can be treated as independent of other time-varying and area-specific factors that might affect health care utilisation and medical expenditure outcomes [19]. Third, our IV estimates show that the introduction of the NRCMS appears to increase the likelihood of people seeking care from village clinics while reducing the usage of informal care providers. Further subgroup analyses suggest that the increase in the use of village clinics is mainly driven by low-income patients while wealthier patients tend to use city hospitals less. To some extent, the insurance provides important and necessary changes to the current health care system, and potentially improve system efficiency by directing patients to primary care health facilities and helping them to obtain more convenient and less costly health services, thus freeing up (more expensive) secondary care services that concentrate on more complex services. The reductions in the use of traditional Chinese folk doctors indicate further benefits of the insurance coverage to prevent people from obtaining health services of insufficient quality.

## Institutional background

The collapse of China’s rural health system in the late 1970s led to unaffordable health care and major financial risks associated with large out-of-pocket health payments [20]. To improve health care access for rural residents, the government established the New Rural Cooperative Medical Scheme (NRCMS). The programme was rolled out across rural counties on a staggered basis: it only covered 10% of rural counties in 2003 and made rapid progress in increasing coverage to very high levels during the next few years. By the end of 2008, the insurance programme was rolled out across the whole country and covered over 90% of the rural population. A previous study that explored the roll-out process using the CHNS dataset found that less urbanised villages appeared to introduce the insurance programme at an earlier point [19]. The eligibility requirement is that people should hold rural residential status, a residential registration scheme designating a citizen as rural or non-rural. All eligible individuals are accepted into the scheme, irrespective of their health status. Although the enrolment is voluntary, most counties achieved a very high participation rate. Figures from National Bureau of Statistics of China show that in 2014, 736 million people were enrolled in the NRCMS, accounting for 99% of the whole rural population [21].

The insurance premium is financed by flat-rate household contributions and government subsidies. All insured individuals within the same county are offered the same premium regardless of their health status and income levels. The benefit package of the NRCMS varies geographically because county administrators are empowered to design their own insurance packages based on local needs and resources. In earlier years, the NRCMS primarily focused on inpatient care for the purpose of protecting people from catastrophic diseases [22], and the programme in all counties covered a specific proportion of inpatient care. As the budget increased, the insurance benefits were also expanded to outpatient services and primary care [23]. Meanwhile a combination of deductibles, co-payments and spending caps are used to control the total health expenditure. On average, the reimbursement rate was targeted at 50% in 2009 [24], increased to 70% in 2011, and 75% in 2015 [25, 26].

Rural China has a three-tiered health system. The bottom tier consists of village clinics that provide basic outpatient services, emergency first aid, immunizations and public health surveillance [10]. Township health centres are the system’s middle tier and provide both outpatient and inpatient services, as well as referral services to higher-level hospitals. The top tier is county, city and higher-level hospitals that provide relatively expensive and specialised inpatient and outpatient medical care. These hospitals are usually located at some distance from villages geographically, and so their use also involves high transportation and other related costs. To restrict overall medical expenditure, the NRCMS encourages patients to seek care from low-level health facilities by providing more generous reimbursement for health services delivered by primary care facilities compared with high-level hospitals [5, 18]. In parallel with the expansion of the NRCMS, the government also aimed at improving the quality and delivery of health services and strengthening pharmaceutical governance in rural areas [27]. Government funding was directed towards improving primary care infrastructure, training general practitioners and integrating village clinics and township health centres into the healthcare delivery system [28]. Primary care providers are expected to play a central part in prevention, case detection and management, gate-keeping and referral [28]. However, recent studies pointed out that some village clinics and township health centres in rural areas still cannot effectively diagnose and treat common diseases, and patients tend to bypass these low-level facilities and seek care at high-level hospitals, even when their symptoms are not indicative of a serious condition [14, 15]. Insufficient clinical knowledge, lack of necessary equipment and poor infrastructure at primary care level remain to be challenging issues and may dampen the effect of expanded access to health care on population health.

## Data

This paper uses panel data from the CHNS, collected by the Carolina Population Centre at the University of North Carolina at Chapel Hill and the National Institute for Nutrition and Health at the Chinese Centre for Disease Control and Prevention. It deployed a multistage, random cluster sampling approach to collect information about key public health risk factors, health outcomes, demographics and socio-economic factors at the individual, household and community levels [29]. The dataset was not designed to be representative of China but to provide data from randomly selected households in nine of China’s 31 provinces, accounting for nearly 44% of China’s total population. Data were collected for ten rounds so far, including waves in 1989, 1991, 1993, 1997, 2000, 2004, 2006, 2009, 2011 and 2015.

This study exploits the longitudinal nature of the dataset from 2000 to 2015, covering the period both before and after the introduction of the NRCMS in 2003. The waves before 2000 are dropped as the survey design and variable information are different from recent waves. We keep only people who lived in rural areas and held a rural residential permit called *hukou*. Five counties are identified as having pre-existing insurance programmes before they launched the NRCMS and are excluded from our sample to reduce bias that might arise from differences in baseline characteristics. Our final sample has 12,190 individuals from 37 rural counties.

The dependent variables in our empirical model include the use of different types of health services (formal medical care, preventive care, folk doctor and inpatient care), different levels of health facilities (village clinics, township health centres, county hospitals and city hospitals) and the total monthly out-of-pocket (OOP) payments (a sum of OOP payment for self-medication, OOP medical payments spent in formal medical facilities, OOP non-medical payments related with health care-seeking behaviour). Note that the recall period for formal medical care, inpatient care, health facility use and OOP payments is 4 weeks; while, it extends to 12 months for the use of preventive care and folk doctors. OOP payments for self-medication and treatment costs in formal medical facilities are calculated based on total medical expenses net of insurance reimbursement. All non-medical expenditure related to care-seeking behaviour, such as transport expense, food cost and accommodation fees, is paid out-of-pocket.

The key independent variable is enrolment in the NRCMS. It is based on the survey question: “what type of medical insurance do you have?” The survey questions in 2004 and 2006 only provide information on whether respondents were enrolled in a cooperative medical scheme (CMS), but do not distinguish between the old CMS and the NRCMS. Following the method used by Lei and Lin [9], we identify whether the village introduced the NRCMS based on the implementation dates of the CMS at village level. NRCMS villages are defined as those that launched the NRCMS after 2003, and an individual is insured if he/she reported to be enrolled in the CMS and was living in an NRCMS village. However, we also note some inconsistency in the introduction years reported by health workers in the same village across different waves, which leads us to cross-validate the implementation dates with another question in the community survey: “Does this village/neighbourhood have CMS in the current wave?” The insured village is identified when health workers reported “yes” for the CMS in any wave after 2003 and “no” in the previous waves. This approach reduces recall bias since health workers should have a better idea about whether the insurance was currently available or not compared with reporting the exact implementation dates. The NRCMS enrolment is defined at household level, which equals one if household heads reported being enrolled. The reason for using this variable is that some household members may be unaware of their insurance status if the head of household is the only one who made the enrolment decision. There are two types of untreated people: non-participants (people who live in NRCMS counties and choose not to enrol) and non-exposed (people who live in non-NRCMS counties and do not have the option of enrolment). We only include non-exposed people in our comparison group since the non-participants may select themselves into the programme based on unobservables that change over time [5]. They may deliberately choose not to enrol because they are confident of their health status and do not need any health services in the near future. Comparing the outcomes of participants with non-exposed people should reduce the selection bias [5]. Other independent variables consist of demographic, socio-economic and health-related characteristics at individual- and household-level, including age, gender, household size, marital status, ethnicity, geographic dummies (eastern, central or western regions), household income, asset index, education level (illiterate, finished primary school, finished junior high school and finished senior high school or above), occupation (farmers and other occupations), number of major diseases (including hypertension, diabetes, heart diseases, apoplexy and bone fracture), severity of illness in the last month and health risk variables (overweight, smoking and daily drinking). We have also controlled for the urbanicity level of the communities as well as area and year fixed effects. The summary statistics of all variables are included in Appendix A.

## Methodology

We start with a cross-sectional logit model to estimate the effect of participating in the NRCMS on medical care utilisation and OOP payments (results are presented in Appendix B):

1 $$Y_{it} = F\left( {\alpha_0 + \alpha_1 {\text{NRCMS}}_{it} + \alpha_2 X_{it} + u_{it} } \right),$$

where ${Y}_{it}$ is the measure of health care utilisation in year *t* for individual *i*. The function $F$ takes the form of a logit function for health care utilisation. Since medical expenditure is positively skewed with a large fraction of the sample at zero and a small number of individuals recording very high values, we account for the OOP distribution using a two-part model on both the probability of making any payment and on the positive amount paid. The former is modelled as a logit, while the latter is estimated using a generalised linear model (GLM) with a Gaussian distribution and an identity link that is applied to the log transformed OOP payments. ${\mathrm{NRCMS}}_{it}$ is an indicator of whether a household is enrolled in the NRCMS or not; ${X}_{it}$ is a set of independent variables as detailed above. ${u}_{it}$ are the error term of mean zero and assumed to be uncorrelated with the regressors ${X}_{it}$. To take into account of the likely autocorrelation in the error structure due to the panel nature of the data, we cluster the standard errors by county, given that the insurance is implemented at a county level. In addition to the effect of the NRCMS on overall population, we also conduct subgroup analysis by income as part of our main analyses.

Self-selection problems may exist if the insured and uninsured groups differ in key aspects that are not observed by researchers prior to insurance coverage in ways that matter for the outcomes studied. As the NRCMS is offered on a voluntary basis, those who are covered may be more likely to be sick or utilise health services and therefore tend to benefit more from the NRCMS compared with those who are not covered. Therefore, simply comparing the mean outcomes of the insured and uninsured people may provide biased estimates. This is a common challenge arising in programme evaluation if the treatment assignment is not random.

To account for unobserved confounding variables, we use an IV approach. Following previous literature on impact evaluation of the NRCMS [9, 23, 30], insurance availability at the county level is used to derive an instrument for individual enrolment into the NRCMS. This is considered an appropriate IV because it satisfies the two requirements of validity: (1) there is a high correlation between the county NRCMS status and household insurance enrolment status, as shown in Tables 1, 2 where the coefficients of county NRMCS status in the first-stage regression are very large, and as we show later, these pass standard thresholds for detecting weak instruments; (2) the roll-out of the NRCMS across counties and over time can be treated as good as random conditional on community characteristics. Previous work has shown that communities located in more deprived areas are more likely to be an early adopter of the NRCMS [19]. For this reason, we have controlled for the urbanicity level of the community, to account for local socio-economic differences that might exist between areas that introduced the NRMCS and areas that did not. We also exploit the longitudinal nature of the data and include province fixed effects and time fixed effects (FE) to capture permanent differences across provinces in unobserved characteristics, and also include secular year-specific effects common to all. Conditioning on the village-level urbanicity index and province fixed effects, we argue that county-level insurance status provides a source of exogenous variation in individual insurance enrolment and it is not likely to be correlated with individual health care utilisation and medical expenses in any other way besides through the insurance. We employ a two-stage least squared regression (2SLS) model: In the first stage, we estimate the probability of household enrolment in the NRCMS as a function of individual and household characteristics and the insurance availability at county level. The estimated probability of being insured will then be used in a second equation to estimate the impact of the insurance on the outcomes. Subject to the validity of the exclusion restriction and instrument relevance, this approach will generate unbiased estimates of the impact of insurance on outcomes of interest. We present the fixed effects IV (FE-IV) estimators from the linear probability model (LPM) and Ordinary least squares (OLS) for the ease of interpretation, although it might give results outside of the zero–one bounds in some cases. We explore the robustness of our results to estimating non-linear FE-IV models.

## Results

Table 1 reports IV estimates for the overall sample (top panel), then additional estimates (bottom panel) where we split the sample by the tercile of household income (the richest 33.3% quantile, the middle 33.3% quantile and the poorest 33.3% quantile). The list of control variables are included as notes below the table, but are not reported on for the sake of brevity. The full regression results for all independent variables can be found in Appendix C. The instrument passes standard tests of instrument weakness in all cases.^1^ After controlling for the province fixed effects and urbanicity level of the community, it is reasonable to assume that the introduction of the NRCMS in a particular county is not related to the underlying health conditions or health care utilisation patterns of the individuals. We find weakly statistically significant reductions in the use of folk doctors, which can be seen as a substitute for formal health care. In terms of facility use, there is a marked increase in the use of village clinics, at a level of 14 percentage point and significant at 5% level. There appears to be a substitution away from more expensive city hospitals to village clinics given a large negative effect on city hospital use that just misses statistical significance at 10% level. This may reflect that the insurance scheme changes health behaviours and alleviates health-related financial difficulties of poorer households. To explore this, we again re-estimate the IV models split according to the household income level. These estimates provide a more nuanced set of results to those pooled across all households, although one has to be careful in interpreting these results as the estimates become imprecise in the sub-sample analyses. For instance, the negative and significant effect of the NRCMS on the use of city hospitals seems to be driven by those who are from the richest income quantile. This may reflect the fact that city hospitals, due to both direct and indirect costs, are only a treatment option for wealthier households in the absence of insurance. The introduction of the NRCMS leads wealthier households to change their treatment-seeking behaviour away from these facilities towards township health centres, indicating that the NRCMS may redistribute utilisation from high-level facilities towards primary care facilities among the better off. The poorest households are more likely to use village clinics with the NCRMS, and again there is a suggestion that this reflects substitution away from all other facility usage.

Table 2 presents IV estimates for OOP expenditures, where again we provide estimates split by income groups of the households. We report these estimates in two parts, first the probability of incurring OOP expenditure, then the effect on the magnitude of these payments conditional on them being incurred. There is a substantial decrease in OOP payment levels among low-income people. As suggested by Table 1, the results here indicate  that the switch from high-level hospitals to primary care facilities might reduce OOP payments, and the effect is more profound for the poor.

**Table 1:** Impact of the NRCMS on medical care utilisation (IV analysis)

	Formal care	Preventive care	Folk doctor use	Inpatient care	Village clinics	Township health centres	County hospitals	City hospitals
**Overall effects**								
NRCMS treatment effect	− 0.001 (0.022)	0.008 (0.013)	− 0.036* (0.021)	0.029 (0.036)	0.144** (0.062)	− 0.043 (0.085)	0.013 (0.053)	− 0.074 (0.046)
F statistics for weak identification test	392.790	401.956	427.834	301.553	318.679	318.679	318.679	318.679
* N*	20,324	20,431	17,322	2056	2042	2042	2042	2042
**Subgroup analyses by income**								
High income	0.001 (0.030)	0.005 (0.015)	− 0.048 (0.035)	0.021 (0.036)	0.083 (0.080)	0.196* (0.106)	0.042 (0.067)	− 0.104** (0.048)
* N*	6627	6652	5688	663	612	612	612	612
Middle income	0.008 (0.025)	0.010 (0.015)	− 0.031 (0.024)	− 0.070 (0.050)	− 0.028 (0.116)	− 0.107 (0.136)	− 0.017 (0.085)	− 0.060 (0.073)
* N*	6886	6933	5869	663	653	653	653	653
Low income	− 0.013 (0.030)	0.010 (0.019)	− 0.026 (0.026)	0.083* (0.048)	0.300*** (0.108)	− 0.195* (0.112)	− 0.040 (0.084)	− 0.046 (0.075)
* N*	6811	6846	5765	783	777	777	777	777

Notes: Results from linear probability model (LPM) controlling for the province and year dummies. Robust standard errors clustered at county level in brackets.

Other independent variables include age, gender, household size, marital status, ethnicity, eastern region, central region, household income, asset index, education level, occupation, number of major diseases, severity of illness in the last month, health risk variables and urbanicity index at community level.

Income quintile groups are computed on the basis of the total equalised disposable income attributed to each member of the household. We divide the sample population into three groups equally represented by 33.33% of the total population each, with two quintile cut-off points.

*Indicates statistical significant at the 10% level; **indicates statistical significant at the 5% level; ***indicates statistical significant at the 1% level.

**Table 2:** Impact of the NRCMS on medical expenditure (IV analysis)

	Two-part model	
	Pr (OOP > 0)	Log of OOP if positive
**Overall effects**		
NRCMS treatment effect	0.032 (0.081)	− 0.320 (0.277)
F statistics for weak identification test	413.315	323.614
* N*	3136	2389
**Subgroup analyses by income**		
High income	0.036 (0.075)	0.071 (0.279)
* N*	916	715
Middle income	0.013 (0.092)	− 0.134 (0.505)
* N*	1011	768
Low income	0.030 (0.131)	− 0.904** (0.391)
* N*	1209	906

Notes: Results from LPM models on the probability of incurring any positive OOP payments and ordinary least square (OLS) models on the log transformed expenditure outcomes controlling for the province and year dummies. Robust standard errors clustered at county level in brackets.

Other independent variables include age, gender, household size, marital status, ethnicity, eastern region, central region, household income, asset index, education level, occupation, number of major diseases, severity of illness in the last month, health risk variables and urbanicity index at community level.

Income quintile groups are computed on the basis of the total equalised disposable income attributed to each member of the household. We divide the sample population into three groups equally represented by 33.33% of the total population each, with two quintile cut-off points.

^**^Indicates statistical significant at the 5% level.

In Table 3, we report a number of checks showing that our results are robust to various alternative specifications. First, we carry out over-identification tests using an additional IV on the number of years since the county first introduced the NRCMS. We hypothesise that expectations about the NRCMS would be shaped by the familiarity about the insurance programme, so that households living in counties with a longer history of insurance coverage are more likely to get enrolled and benefit from the insurance. Thus, we use the length of insurance coverage as another instrument for household enrolment. The instruments pass the joint test of validity and are shown to be uncorrelated with the error terms. There is also a strong effect of the instruments on the household enrolment in the insurance, based on the first-stage F statistics. Therefore, the testing results give us a good deal of confidence in our instruments. We find that both the point estimates and significance level are nearly identical to the main results in Tables 1, 2, except that the increase in the use of village clinics becomes significant only at 10% level. Notice that there might be some noise in the reported NRCMS inception dates given that a considerable number of respondents who report the year inconsistently across waves. The measurement errors in the IV variable might lead to a bias in the estimates here. In the second panel of Table 3, we present the marginal effects of the NRCMS from IV probit models to account for the binary nature of the health utilisation outcomes and censored OOP payments. We have calculated the marginal effects at the mean values of the NRCMS regressor. When compared with earlier estimates, we obtain even larger and statistically significant effects on the use of folk doctors and village clinics. For medical expenditure, we further experiment with sample selection model and Tobit model, and the results appear similar to those in Table 2 (Appendix D).

**Table 3:** Robustness checks on the impact of the NRCMS on health care utilisation and medical expenditure

	Formal care	Preventive care	Folk doctor use	Inpatient care	Village clinics	Township health centres	County hospitals	City hospitals	Pr(OOP > 0)	Log of OOP if positive
**(1) IV of county NRCMS status and number of years of the NRCMS coverage**										
NRCMS treatment effect	− 0.000 (0.022)	0.005 (0.014)	− 0.037* (0.022)	0.024 (0.034)	0.116* (0.068)	− 0.029 (0.085)	0.016 (0.054)	− 0.077* (0.046)	0.023 (0.082)	− 0.298 (0.279)
F statistics for weak identification test	242.467	248.267	262.863	166.822	190.580	190.580	190.580	190.580	251.227	183.438
Sargan’s over-identification test (prob)	0.753	0.127	0.718	0.208	0.303	0.113	0.288	0.594	0.796	0.597
* N*	17,397	17,467	14,361	1754	1744	1744	1744	1744	2688	2021
**(2) Non-linear IV models**										
NRCMS treatment effect	− 0.024 (0.118)	0.188 (0.243)	− 0.443** (0.219)	0.218 (0.321)	0.418** (0.206)	− 0.185 (0.301)	0.019 (0.233)	− 0.603 (0.372)	0.073 (0.249)	− 0.320 (0.277)
* N*	20,324	20,431	17,322	2048	2042	2042	2012	1932	3136	2389

Notes: The first panel shows results from IV estimations in linear probability models (LPM) using 2 IVs: the introduction of the NRCMS at county level and the number of years covered by the insurance, with F statistics for weak identification test and probability of Sargan’s over-identification test. Sargan’s over-identification test is calculated as N*R-squared from a regression of the IV residuals on both instruments. The joint null hypothesis is that the instruments are valid instruments; a rejection casts doubt on the validity of the instruments. The second panel shows the IV probit estimation results for health care utilisation. Two different models are applied to the two parts of the OOP payments: an IV probit model for the probability that an individual made any OOP payment and OLS, applied only to sub-sample with non-zero OOP payments, for the log of OOP payments. Robust standard errors clustered at county level in brackets.

Other independent variables include age, gender, household size, marital status, ethnicity, eastern region, central region, household income, asset index, education level, occupation, number of major diseases, severity of illness in the last month, health risk variables and urbanicity index at community level.

^*^Indicates statistical significant at the 10% level; ^**^indicates statistical significant at the 5% level.

In Table 4, we perform further subgroup analyses by region. Regional variation in health and health care has long been a policy concern in China. In particular, we are interested to see whether individuals from deprived regions tend to gain more from the insurance as this could contribute to closing the geographic gaps in health—one of the principle aims of the national health care reform. We can see that the increase in the use of village clinics is mainly driven by people from central and western regions, although the point estimate is not statistically significant for people living in the west. This is mainly due to the small sample size, leading to a relatively large standard error. The magnitude of the point estimate, however, is much larger than the one found in the pooled sample. At the same time, there is a substantial decline in the use of city hospitals in western China, although it is only significant at the 10% level. The result is accompanied by significant increases in the probabilities of incurring any OOP payments among eastern and western residents, while substantial although insignificant decreases in the levels of OOP payments across all regions. It appears that insured patients are less likely to forego health care, but it does not necessarily lead to larger medical payments due to a switch from high-level hospitals to village clinics. In line with the key objectives of the insurance programme, patients from more deprived western provinces seem to benefit more from the NRCMS.

**Table 4:** Subgroup analysis of the impact of the NRCMS on medical care utilisation by region

	Formal care	Preventive care	Folk doctor use	Inpatient care	Village clinics	Township health centres	County hospitals	City hospitals	Pr (OOP > 0)	Log of OOP if positive
**Subgroup analysis by regions**										
Eastern provinces	− 0.012 (0.019)	− 0.009 (0.019)	− 0.076^**^ (0.031)	− 0.006 (0.086)	0.029 (0.164)	0.022 (0.184)	− 0.081 (0.195)	0.078* (0.046)	0.159*** (0.050)	− 0.057 (0.504)
* N*	4757	4808	4077	362	359	359	359	359	654	518
Middle provinces	− 0.007 (0.028)	0.001 (0.018)	− 0.033 (0.023)	0.010 (0.048)	0.224*** (0.058)	− 0.065 (0.104)	− 0.001 (0.066)	− 0.086 (0.053)	− 0.119 (0.088)	− 0.418 (0.331)
* N*	10,239	10,269	8748	1090	1082	1082	1082	1082	1593	1195
Western provinces	0.039 (0.036)	0.062 (0.042)	− 0.034 (0.046)	0.092* (0.050)	0.197 (0.189)	− 0.193 (0.230)	0.136 (0.096)	− 0.164* (0.098)	0.452*** (0.175)	− 0.334 (0.343)
* N*	5328	5354	4497	604	601	601	601	601	889	676

Notes: Results from linear probability model (LPM) controlling for the province and year dummies. Robust standard errors clustered at county level in brackets.

Other independent variables include age, gender, household size, marital status, ethnicity, household income, asset index, education level, occupation, number of major diseases, severity of illness in the last month, health risk variables and urbanicity index at community level.

*Indicates statistical significant at the 10% level; **indicates statistical significant at the 5% level; ***indicates statistical significant at the 1% level.

## Discussion and conclusion

This study is one of the few analyses to examine the long-term effects of expanding social health insurance among people working in informal sectors. It shows that the effects of a government-funded insurance policy in a context where the majority of people remained uninsured prior to the introduction of the programme and paid health care services mainly out-of-pocket. These conditions are likely to hold in many other low- and middle-income countries that are considering whether to invest more in national insurance schemes to achieve universal coverage. To our knowledge, this study is unique in that it considers the effects of the introduction of a social health insurance programme that covers such a large population group over a long period of time. This helps to inform the debate on the potential benefits of the social health insurance programme to increase the access to health care and offer financial protection to individuals and households, and provides valuable insights on how to design the policy in an effective way.

While our results are generally in line with previous studies that suggest limited effectiveness of the NRCMS on overall medical care use and OOP payments in rural China [9–11], this overall lack of effect masks variation in the impact of insurance on different types of health services and health providers, as well as across different population groups. We find that the NRCMS appears to encourage patients to switch from city hospitals to village clinics. Further subgroup analyses by income and region suggest that wealthier individuals tend to reduce their use of city hospitals, while the increase in village clinic use mainly occurs among people with lower household income or from more deprived western areas.

The substitution across different levels of health facilities indicates that the NRCMS may have partly corrected any distortion in rural health care system by promoting low profit margin primary services and reducing the use of specialty services at high-level health facilities. The results are consistent with previous work that also found an increase in the use of village clinics or township health centres after the insurance was introduced [5, 10, 11]. The expansion of the insurance benefits to outpatient services in recent years provides incentives for patients to substitute hospital services with primary care, particularly among poor people. However, we cannot exclude the possibility that this result might be driven by insufficient finances, given that the majority of people affected come from disadvantaged backgrounds. They might only be able to afford care delivered by village clinics and have to prioritise price over quality of care when choosing place for treatments [8]. We could also find some clues from the fact that the better off tend to switch to township health centres rather than village clinics with insurance coverage, indicating their reluctance to compromise quality of care for insurance benefits. In addition, since village clinics and township health centres seldom provide inpatient care, costs incurred at these facilities might be reimbursed at a very low level [31]. This could be one of the reasons why there is limited effects of the NRCMS on OOP payments, even if insured patients switch to less costly primary care services. Previous work has shown that household spending on outpatient care sometimes can also cause medical impoverishment, especially among the poor [32]. Therefore, the government’s goal of achieving financial protection through social health insurance may not be realistic if the reimbursement for outpatient expenditure remains low.

As with all IV models, one must be cautious about instrument validity. Our identifying assumption is that the IV—insurance availability at county level—is not correlated with health utilisation and medical expenses in any way other than through its effect on insurance. Although this assumption is supported by previous evidence, it might still be subject to criticism.

In summary, the findings from this study add new evidence to the long-term impact of the NRCMS shedding some light on the mechanism that drives the effects. The results indicate some degree of success of the NRCMS in improving the efficiency within the health care system and reducing the use of health care from informal providers and self-medication. The programme appears to have met one of its key goals of directing patients to seek care from primary care facilities and rationalising the use of health services. At the same time, the shift from self-medication and folk doctors to better regulated formal medical providers is likely to improve safety and quality of care. However, the overall benefits of insurance coverage on health care utilisation and OOP payments appear to be rather modest. The increase in the use of village clinics among people with lower socio-economic status indicates that they may be constrained by limited financial ability to access costly treatments for severe and complex conditions from high-level facilities. Unless the generosity of insurance reimbursements can be sufficiently expanded to outpatient services at primary care level, the initial benefits from the NRCMS might fade out gradually over time, given that village clinics are the only reasonable source of medical care for many rural people from remote villages [10, 33]. If universal coverage is to be achieved through the expansion of the social health insurance, as in our case, it is important to monitor and enforce high quality standards for primary care services and provide sufficient coverage through insurance reimbursements. The policy implications might be useful for other countries that are in the process of reforming their health care systems and expanding social health insurance schemes to a wider population.

## Supplementary Information

Below is the link to the electronic supplementary material.

Supplementary file1 (DOCX 189 KB)

## Footnotes

1 We do not report the F statistics for weak identification test for subgroup analyses, but in all cases, the instrument easily passes the standard threshold for detecting weak instruments.
