# Supplementary material for: The impact of social health insurance on rural populations
Source: Eur J Health Econ. 2021 Feb 27;22(3):473–83. doi: 10.1007/s10198-021-01268-2 (PMC7954739; doi:10.1007/s10198-021-01268-2)
Supplement: Supplementary file 1 — Supplementary file1 (DOCX 189 KB) [file 10198_2021_1268_MOESM1_ESM.docx]

**Appendix A: Summary statistics**

Table A1 presents the summary statistics of all dependent and independent variables by insurance status included in the analysis. Overall, there is no significant difference between the insured and uninsured groups in the mean level of most health care use and medical expenditure outcomes, although the insured appeared to use more village clinics compared with those uninsured.

In Table A2, we provide a comparison of individual-, household- and community-level characteristics between treatment and comparison groups. This is a preview of the data included as independent variables in the empirical model. We find that the treatment group is quite different from the comparison group: the treated have on average more favourable socio-economic conditions (e.g. higher education levels and employment rates, earn more income and possess more household assets, less likely to be a farmer) and live in more urbanized communities. However, in terms of the health-related characteristics, the insured individuals tend to have more major diseases, be overweight, more likely to be a smoker and alcohol drinker.

**Table A1: Summary statistics of dependent variables**

|  | **Insured^a^**  **(n=16,939)** | | **Uninsured**  **(n=12,901)** | | **Mean difference (95% confidence interval)^b^** |
| --- | --- | --- | --- | --- | --- |
|  | N | Mean % (SD) | N | Mean % (SD)^c^ |  |
| Formal medical care | 1,999 | 11.95(0.32) | 1,020 | 8.02(0.27) | *-0.00(-0.03, 0.03)* |
| *Unknown* | *215* |  | *186* |  |  |
| Preventive care | 544 | 3.22(0.18) | 150 | 1.19(0.11) | *0.01(-0.02, 0.03)* |
| *Unknown* | *19* |  | *266* |  |  |
| Folk doctor utilization | 852 | 5.04(0.22) | 293 | 4.27(0.20) | *-0.03(-0.06, 0.01)* |
| Unknown | *38* |  | *6,037* |  |  |
| Inpatient care | 195 | 9.91(0.30) | 55 | 5.80(0.23) | *0.03(-0.02, 0.09)* |
| *Unknown* | *14,972* |  | *11,953* |  |  |
| Village clinics | 766 | 39.44(0.49) | 303 | 32.17(0.47) | *0.12(0.03, 0.21)* |
| *Unknown* | *14,997* |  | *11,959* |  |  |
| Township health centres | 471 | 24.25(0.43) | 244 | 25.90(0.44) | *-0.06(-0.17, 0.06)* |
| *Unknown* | *14,997* |  | *11,959* |  |  |
| County hospitals | 250 | 12.87(0.33) | 121 | 12.85(0.33) | *0.00(-0.07, 0.07)* |
| *Unknown* | *14,997* |  | *11,959* |  |  |
| City hospitals | 139 | 7.16(0.26) | 75 | 7.96(0.27) | *-0.04(-0.10, 0.03)* |
| *Unknown* | *14,997* |  | *11,959* |  |  |
| Total OOP payments during the last 4 weeks | 2,254 | 831.84(5,093.27) | 1,115 | 350.41(1,520.46) | *184.46(-204.97, 573.89)* |
| *Unknown* | *14,000* |  | *11,378* |  |  |

*Notes*: ^a^ The insured are households covered by the NRCMS and currently living in NRCMS counties. The uninsured are households who lived in non-NRCMS counties. We exclude households who lived in NRCMS counties but chose not to get enrolled, and households who were covered by any other insurance programme besides the NRCMS. ^b^ Adjusted for wave and province dummies and clustering with 95% confidence intervals used; Difference in mean proportions for dummy variables and difference in sample means for continuous variables;  ^c^ Mean in proportion for dummy variables, and sample mean for continuous variables; SD: standard deviation.

**Table A2: Comparison of baseline characteristics**

|  | **Insured**  **(n=16,939)** | **Uninsured**  **(n=12,901)** | **p-value^c^** |
| --- | --- | --- | --- |
| **Individual-level characteristics** | | | |
| Age, Median(IQR) | 45.00(26.00,58.00) | 37.00(19.00,51.00) | 0.000^***^ |
| *Unknown* | *0* | *3* |  |
| Female, n (%) | 8,739(51.59%) | 6,464(50.10%) | 0.011^**^ |
| *Unknown* | *0* | *0* |  |
| Marital status, n (%) | 11,739(69.30%) | 7,825(60.65%) | 0.000^***^ |
| *Unknown* | *3,272* | *1,648* |  |
| Illiterate, n (%) | 3,408(20.12%) | 3,325(25.77%) | 0.000^***^ |
| Primary, n (%) | 3,912(23.09%) | 3,515(27.25%) |  |
| Junior high school, n (%) | 5,337(31.51%) | 4,058(31.45%) |  |
| Senior high school and above, n (%) | 1,582(9.34%) | 990(7.67%) |  |
| *Unknown* | *2,700* | *1,013* |  |
| Working, n (%) | 8,591(50.72%) | 7,746(60.04%) | 0.000^***^ |
| *Unknown* | *3,077* | *2,483* |  |
| Farmer, n (%) | 5,860(34.59%) | 5,880(45.58%) | 0.000^***^ |
| *Unknown* | *3,077* | *2,462* |  |
| Number of major diseases, Mean(SD) | 0.14(0.40) | 0.06(0.27) | 0.000^***^ |
| *Unknown* | *0* | *0* |  |
| Disease severity last month, Mean(SD)^a^ | 1.67(0.65) | 1.68(0.68) | 0.492 |
| *Unknown* | *14,254* | *11,500* |  |
| Overweight, n (%) | 6,602(38.98%) | 3,286(25.47%) | 0.000^***^ |
| *Unknown* | *1,380* | *2,025* |  |
| Smoking, n (%) | 3,867(22.83%) | 2,775(21.51%) | 0.008^***^ |
| *Unknown* | *2,503* | *3,106* |  |
| Alcohol daily drinker, n (%) | 1,245(7.35%) | 857(6.64%) | 0.783 |
| *Unknown* | *2,673* | *3,195* |  |
|  |  |  |  |
| **Household-level characteristics** | | | |
| Household size, Median(IQR) | 4.00(3.00,5.00) | 4.00(3.00,5.00) | 0.001^***^ |
| *Unknown* | *250* | *164* |  |
| Minority ethnicity, n (%) | 2,908(17.17%) | 2,561(19.85%) | 0.00^***^ |
| *Unknown* | *333* | *401* |  |
| Eastern provinces, n (%) | 3,869(22.84%) | 2,370(18.37%) | 0.00^***^ |
| Middle provinces, n (%) | 8,381(49.48%) | 6,851(53.10%) |  |
| Western provinces, n (%) | 4,689(27.68%) | 3,680(28.52%) |  |
| Household income, Median(IQR) | 28,000.00(13,441.56,52,150.54) | 13,812.87(6,938.60,24,048.67) | 0.00^***^ |
| *Unknown* | *249* | *153* |  |
| Asset index, Median(IQR) | 0.28(-0.72,1.31) | -1.51(-2.28,-0.63) | 0.00^***^ |
| *Unknown* | *1* | *0* |  |
|  |  |  |  |
| **Community-level characteristics** | | | |
| Urbanicity index, Median(IQR) ^b^ | 54.22(44.09,62.08) | 44.58(36.99,52.72) | 0.000^***^ |
| *Unknown* | *0* | *0* |  |

*Notes*: ^a^ Disease severity level: 1 not severe; 2 somewhat severe; and 3 very severe. ^b^The urbanicity level of the village was defined based on Jones-Smith and Popkin (2010), which is the sum of 12 components that are used to define and distinguish the urbanicity level of a community, including population density, economic activity, traditional markets, modern markets, transportation infrastructure, sanitation, communications, housing, education, diversity, health infrastructure and social services at community level. ^c^ ^**^ indicates statistical significant at the 5% level; ^***^ indicates statistical significant at the 1% level.

**Appendix B: Cross-sectional estimates on the impact of the NRCMS on health care utilization and medical expenditure**

Table B1 presents logit estimation of the relationship between NRCMS coverage and health care utilization. To aid interpretation, we report the coefficients of interest as marginal effects. In terms of health care utilization, the main apparent patterns are a small increase in the use of preventative care (1.8 percentage points), and an increase in inpatient care usage (3.5 percentage points), mainly driven by low-income households. There are no statistically significant relationship between NRCMS coverage and formal care or folk doctor use. The most dramatic patterns are in terms of the type of facility usage. The use of village clinics is almost 20 percentage points higher among insured people compared with uninsured people, and this pattern is apparent across all income groups. There is some suggestion that this reflects substitution away from other forms of facility usage. The signs of the effects of the NCRMS on the use of township health centres, county and city hospitals are negative, although none are statistically significant at standard levels. To further explore this point, in unreported estimates we pool all of these into one variable (have you used a township health centre, county or city hospital in the last 4 weeks) and NCRMS usage is associated with a 9.2 percentage point reduction in usage of these facilities and this is statistically significant at the 5% level.

Table B2 reports estimates of the effect of NRCMS on medical expenditure. We report these estimates in two parts, first the probability of incurring OOP expenditure, and then the effect on the magnitude of these payments conditional on them being incurred. Again, reflecting the concern that schemes such as the NRCMS have as a goal the alleviation of financial barriers to health care utilization amongst the poor, we provide additional estimate split by household income levels. The headline result is that there is no evidence of an effect of the NRCMS on the overall probability of incurring OOP expenditures, or on their magnitude. No significant difference in the NRCMS effect is found across income groups either.

A key concern with interpreting the estimates in Tables B1 and B2 is that the treated and comparison groups differ prior to treatment in ways that matter for the outcomes under study. As the NRCMS is offered on a voluntary basis, participants and non-participants may differ in terms of unobserved individual characteristics that influence both their decision to participate in the programme and their levels of outcomes. For instance, the insured people may deliberately select themselves into the NRCMS because they expect to use many health services and benefit more from the insurance. Reflecting these types of concerns, the results presented here should only be treated as baseline estimates and please refer to Tables 1 and 2 for estimates that can be interpreted causally.

**Table B1: Impact of the NRCMS on medical care utilization (Logit models)**

|  | Formal care | Preventive care | Folk doctor use | Inpatient care | Village clinics | Township health centres | County hospitals | City hospitals |
| --- | --- | --- | --- | --- | --- | --- | --- | --- |
| **Overall effects** | | | | | | | | |
| NRCMS membership | 0.017^*^ | 0.018^***^ | 0.002 | 0.024^**^ | 0.198^***^ | -0.044 | -0.019 | -0.018 |
|  | (0.010) | (0.006) | (0.008) | (0.011) | (0.047) | (0.036) | (0.019) | (0.017) |
| *N* | 18995 | 19085 | 16023 | 1901 | 1887 | 1887 | 1887 | 1887 |
|  |  |  |  |  |  |  |  |  |
| **Subgroup analysis by income** | | | | | | | | |
| High income | 0.018 | 0.015^**^ | 0.006 | 0.004 | 0.265^***^ | 0.011 | -0.018 | -0.045^**^ |
|  | (0.013) | (0.008) | (0.009) | (0.018) | (0.050) | (0.037) | (0.039) | (0.022) |
| *N* | 6156 | 6177 | 5235 | 562 | 564 | 564 | 564 | 564 |
|  |  |  |  |  |  |  |  |  |
| Middle income | 0.016 | 0.018^***^ | 0.009 | 0.010 | 0.126^*^ | -0.063 | -0.013 | 0.004 |
|  | (0.013) | (0.005) | (0.012) | (0.021) | (0.072) | (0.051) | (0.042) | (0.020) |
| *N* | 6431 | 6471 | 5423 | 613 | 602 | 602 | 602 | 602 |
|  |  |  |  |  |  |  |  |  |
| Low income | 0.019 | 0.014^*^ | -0.002 | 0.032^*^ | 0.168^**^ | -0.069 | -0.022 | -0.003 |
|  | (0.014) | (0.008) | (0.011) | (0.016) | (0.076) | (0.056) | (0.026) | (0.024) |
| *N* | 6408 | 6437 | 5365 | 726 | 721 | 721 | 721 | 721 |

*Notes*:  Marginal effects from logit models. Robust standard errors clustered at county level in brackets. ^*^ indicates statistical significant at the 10% level. ^**^ indicates statistical significant at the 5% level. ^***^ indicates statistical significant at the 1% level. Other independent variables include age, gender, household size, marital status, ethnicity, eastern region, central region, household income, asset index, education level, occupation, number of major diseases, severity of illness in the last month, health risk variables and urbanicity index at community level. Income quintile groups are computed on the basis of the total equalised disposable income attributed to each member of the household. We divide the sample population into three groups equally represented by 33.33% of the total population each, with two quintile cut-off points.

**Table B2: Impact of the NRCMS on medical expenditure (two-part model)**

|  | | **Pr(OOP >0)** | | **Log of OOP if positive** | |
| --- | --- | --- | --- | --- | --- |
| **Overall effects** | |  | |  | |
| NRCMS treatment effect | | 0.056 | | 0.198^*^ | |
|  |  | (0.035) | | (0.117) | |
| *N* | | 2908 | | 2218 | |
| **Subgroup analysis by income** |  | |  | |  |
| High income | | 0.062 | | 0.291^*^ | |
|  |  | (0.054) | | (0.170) | |
| *N* | | 860 | | 669 | |
|  | |  | |  | |
| Middle income | | 0.042 | | 0.336^*^ | |
|  | | (0.051) | | (0.201) | |
| *N* | | 930 | | 711 | |
|  | |  | |  | |
| Low income | | 0.052 | | 0.052 | |
|  | | (0.045) | | (0.184) | |
| *N* | | 1118 | | 838 | |

*Notes*: Marginal effects from logit models for Pr(OOP>0) and from generalised linear models with a Gaussian distribution and an identity link on the log-transformed OOP. Robust standard errors clustered at county level in brackets. ^*^ indicates statistical significant at the 10% level. Other independent variables include age, gender, household size, marital status, ethnicity, eastern region, central region, household income, asset index, education level, occupation, number of major diseases, severity of illness in the last month, health risk variables and urbanicity index at community level. Income quintile groups are computed on the basis of the total equalised disposable income attributed to each member of the household. We divide the sample population into three groups equally represented by 33.33% of the total population each, with two quintile cut-off points.

**Appendix C: Full results of IV estimations**

Tables C1 and C2 present the full results of Tables 1 and 2 with marginal effects for all independent variables included in the models.

**Table C1: Impact of the NRCMS on medical care utilization (IV analysis)**

|  | Formal care | Preventive care | Folk doctors | Inpatient care | Village clinics | Town hospitals | County hospitals | City hospitals |
| --- | --- | --- | --- | --- | --- | --- | --- | --- |
| NRCMS | -0.001 | 0.008 | -0.036^*^ | 0.029 | 0.144^**^ | -0.043 | 0.013 | -0.074 |
|  | (0.022) | (0.013) | (0.021) | (0.036) | (0.062) | (0.085) | (0.053) | (0.046) |
| Age | 0.002^***^ | 0.000^**^ | 0.001^***^ | -0.000 | 0.001 | 0.001 | -0.001^*^ | 0.000 |
|  | (0.000) | (0.000) | (0.000) | (0.001) | (0.001) | (0.001) | (0.001) | (0.001) |
| Female | 0.006 | 0.005^*^ | 0.005 | -0.028^**^ | 0.031 | -0.027 | -0.009 | 0.008 |
|  | (0.006) | (0.003) | (0.005) | (0.011) | (0.029) | (0.023) | (0.015) | (0.014) |
| Married | -0.001 | -0.001 | 0.009^*^ | 0.011 | -0.040^**^ | 0.023 | 0.052^**^ | 0.021 |
|  | (0.007) | (0.003) | (0.005) | (0.021) | (0.018) | (0.024) | (0.024) | (0.015) |
| Illiterate | 0.020^***^ | -0.005 | 0.016^**^ | 0.010 | -0.070 | 0.037 | 0.065^**^ | -0.050^*^ |
|  | (0.008) | (0.007) | (0.007) | (0.024) | (0.046) | (0.044) | (0.028) | (0.026) |
| Primary | 0.006 | -0.005 | 0.010^*^ | 0.021 | -0.042 | 0.057 | 0.066^**^ | -0.057^*^ |
|  | (0.007) | (0.005) | (0.006) | (0.022) | (0.047) | (0.039) | (0.030) | (0.030) |
| Junior | -0.002 | -0.003 | 0.009 | 0.022 | -0.078^*^ | 0.052^*^ | 0.042 | -0.029 |
|  | (0.007) | (0.005) | (0.005) | (0.018) | (0.043) | (0.031) | (0.030) | (0.025) |
| Working | -0.037^***^ | -0.005 | -0.016^**^ | -0.046^**^ | 0.063^*^ | -0.010 | 0.020 | -0.051^***^ |
|  | (0.006) | (0.005) | (0.008) | (0.020) | (0.035) | (0.028) | (0.022) | (0.016) |
| Farmer | 0.001 | 0.000 | 0.006 | 0.029^*^ | 0.014 | 0.023 | -0.066^***^ | 0.021 |
|  | (0.007) | (0.004) | (0.006) | (0.016) | (0.035) | (0.033) | (0.020) | (0.015) |
| No. of major diseases | 0.127^***^ | 0.025^***^ | 0.011^**^ | 0.033^**^ | -0.018 | 0.020 | 0.013 | 0.026^**^ |
|  | (0.010) | (0.005) | (0.004) | (0.016) | (0.014) | (0.021) | (0.016) | (0.010) |
| Overweight | -0.007 | 0.004^*^ | -0.006 | -0.006 | -0.019 | -0.005 | -0.007 | 0.016 |
|  | (0.004) | (0.002) | (0.004) | (0.013) | (0.029) | (0.019) | (0.016) | (0.014) |
| Smoking | -0.005 | 0.000 | -0.000 | -0.026^**^ | -0.010 | 0.011 | -0.041^**^ | 0.026 |
|  | (0.007) | (0.003) | (0.005) | (0.013) | (0.028) | (0.024) | (0.017) | (0.019) |
| Daily drinker | -0.019^*^ | -0.001 | -0.002 | -0.032 | 0.034 | -0.036 | 0.035 | -0.009 |
|  | (0.010) | (0.004) | (0.006) | (0.021) | (0.033) | (0.041) | (0.026) | (0.020) |
| Household size | -0.005^***^ | -0.001 | 0.004^*^ | -0.002 | 0.008 | 0.005 | -0.004 | 0.002 |
|  | (0.002) | (0.001) | (0.002) | (0.004) | (0.009) | (0.007) | (0.008) | (0.005) |
| Minority ethnicity | -0.008 | 0.001 | -0.017^**^ | 0.042^*^ | 0.027 | -0.030 | 0.085^***^ | -0.016 |
|  | (0.011) | (0.003) | (0.007) | (0.022) | (0.054) | (0.047) | (0.026) | (0.024) |
| East | -0.039 | 0.030^*^ | -0.154^**^ | 0.043 | -0.199 | 0.144 | 0.494^***^ | -0.047 |
|  | (0.046) | (0.017) | (0.071) | (0.046) | (0.127) | (0.115) | (0.102) | (0.034) |
| Middle | -0.050 | 0.019 | -0.096 | 0.128^***^ | -0.333^**^ | 0.280^**^ | 0.129^***^ | 0.112^***^ |
|  | (0.037) | (0.014) | (0.077) | (0.042) | (0.135) | (0.130) | (0.037) | (0.042) |
| Household income | 0.003^***^ | 0.002^**^ | 0.004^***^ | 0.007^***^ | -0.004 | -0.004 | 0.009^*^ | -0.000 |
|  | (0.001) | (0.001) | (0.001) | (0.002) | (0.008) | (0.008) | (0.005) | (0.003) |
| Asset index | -0.008^***^ | 0.002 | -0.007^**^ | 0.001 | -0.030^**^ | -0.003 | 0.006 | 0.016^***^ |
|  | (0.003) | (0.002) | (0.003) | (0.005) | (0.012) | (0.011) | (0.007) | (0.005) |
| Urbanicity Index | -0.001^*^ | -0.000 | 0.000 | 0.000 | -0.004^***^ | -0.001 | 0.001 | 0.002^*^ |
|  | (0.000) | (0.000) | (0.000) | (0.001) | (0.001) | (0.002) | (0.001) | (0.001) |
| Disease severity |  |  |  | 0.097^***^ | -0.120^***^ | -0.004 | 0.077^***^ | 0.058^***^ |
|  |  |  |  | (0.011) | (0.014) | (0.016) | (0.014) | (0.014) |
| *N* | 20324 | 20431 | 17322 | 2056 | 2042 | 2042 | 2042 | 2042 |

*Notes*: Results from linear probability model (LPM) controlling for province and year dummies. Robust standard errors clustered at county level in brackets. ^*^ indicates statistical significant at the 10% level. ^**^ indicates statistical significant at the 5% level. ^***^ indicates statistical significant at the 1% level.

**Table C2: Impact of the NRCMS on medical expenditure (IV analysis)**

|  | **Pr(OOP >0)** | **Log of OOP if positive** |
| --- | --- | --- |
| NRCMS | 0.032 | -0.320 |
|  | (0.081) | (0.277) |
| Age | -0.002^***^ | -0.003 |
|  | (0.001) | (0.003) |
| Female | 0.033 | -0.221^**^ |
|  | (0.021) | (0.095) |
| Married | 0.036^*^ | 0.396^***^ |
|  | (0.020) | (0.092) |
| Illiterate | -0.052^*^ | 0.084 |
|  | (0.030) | (0.135) |
| Primary | 0.005 | -0.013 |
|  | (0.030) | (0.144) |
| Junior | -0.036 | 0.173 |
|  | (0.025) | (0.109) |
| Working | 0.065^**^ | -0.471^***^ |
|  | (0.025) | (0.113) |
| Farmer | -0.003 | -0.015 |
|  | (0.023) | (0.111) |
| Number of major diseases | 0.006 | 0.295^***^ |
|  | (0.013) | (0.073) |
| Overweight | 0.013 | -0.057 |
|  | (0.017) | (0.069) |
| Smoking | 0.002 | -0.307^***^ |
|  | (0.023) | (0.100) |
| Daily drinker | 0.001 | 0.117 |
|  | (0.027) | (0.139) |
| Household size | -0.003 | -0.036^*^ |
|  | (0.007) | (0.021) |
| Minority ethnicity | -0.019 | 0.079 |
|  | (0.025) | (0.102) |
| East | 0.030 | 1.085^**^ |
|  | (0.043) | (0.444) |
| Middle | -0.164^***^ | 0.539 |
|  | (0.041) | (0.416) |
| Household income | 0.000 | 0.012 |
|  | (0.006) | (0.020) |
| Asset index | 0.006 | 0.033 |
|  | (0.006) | (0.040) |
| Urbanicity Index | 0.001 | 0.005 |
|  | (0.001) | (0.004) |
| Disease severity | 0.078^***^ | 1.095^***^ |
|  | (0.011) | (0.066) |
| *N* | 3136 | 2389 |

*Notes*: Results from LPM models on the probability of incurring any positive OOP payments and Ordinary least square (OLS) models on the log transformed expenditure outcomes controlling for province and year dummies. Robust standard errors clustered at county level in brackets. ^*^ indicates statistical significant at the 10% level. ^**^ indicates statistical significant at the 5% level. ^***^ indicates statistical significant at the 1% level.

**Appendix D: Robustness checks for medical expenditure**

Table D1 presents two alternative statistical approaches to model OOP payments: sample selection model and Tobit model. To combine the sample selection model with the IV estimations, we first use a probit model for the probability of non-zero OOP payments to estimate the Inverse Mill’s ratios (IMR), conditioning on all exogenous variables and the instrument. In the second stage, we apply IV approach to the non-zero OOP payments with the estimated IMR to correct for selection bias. On the contrary, Tobit model considers OOP payments as a single decision, treating zero expenditure as a corner solution. We use maximum likelihood estimation to fit Tobit model within an IV estimation framework. None of the estimate on OOP payments appears to be statistically significant, which corresponds to the results in Table 2.

**Table D1: Robustness checks on the impact of the NRCMS on medical expenditure**

|  | | | | | **Sample selection model** | | **Tobit model** |  |  |
| --- | --- | --- | --- | --- | --- | --- | --- | --- | --- |
|  | | | | | **Prob(OOP>0) (probit)** | **Log of OOP if positive (OLS)** | **OOP level** |  |  |
|  |  |  |  |  |  |  |  |  |  |
| NRCMS treatment effect | | | | | 0.016 | -0.446 | -165.929 |  |  |
|  |  |  |  |  | (0.033) | (0.277) | (429.097) |  |  |
| *N* | | | | | 3138 | 2389 | 3136 |  |  |

*Notes*: Results from non-linear IV estimation results for medical expenditure. Other independent variables include age, gender, household size, marital status, ethnicity, eastern region, central region, household income, asset index, education level, occupation, number of major diseases, severity of illness in the last month, health risk variables and urbanicity index at community level.

**Appendix E: Placebo tests on the trend of outcome variables over years**

Identification of our IV approach relies on the similarity of the counties that introduced the insurance earlier versus those that introduced at a later point. In this section, we present the descriptive statistics on the outcome variables as early as 1993. Figure E1 shows that most outcome variables follow similar pre-treatment time trends between insured and uninsured individuals. In order to allow for a longer time trend for comparison, we use 2009 as a cut-off point and define the insured group as people who were insured and lived in the NRCMS counties in 2009. The trends based on earlier cut-off points (e.g. 2004 and 2006) show similar patterns.

**Figure E1: Outcome trends over years in insured and uninsured counties in 2009**

**
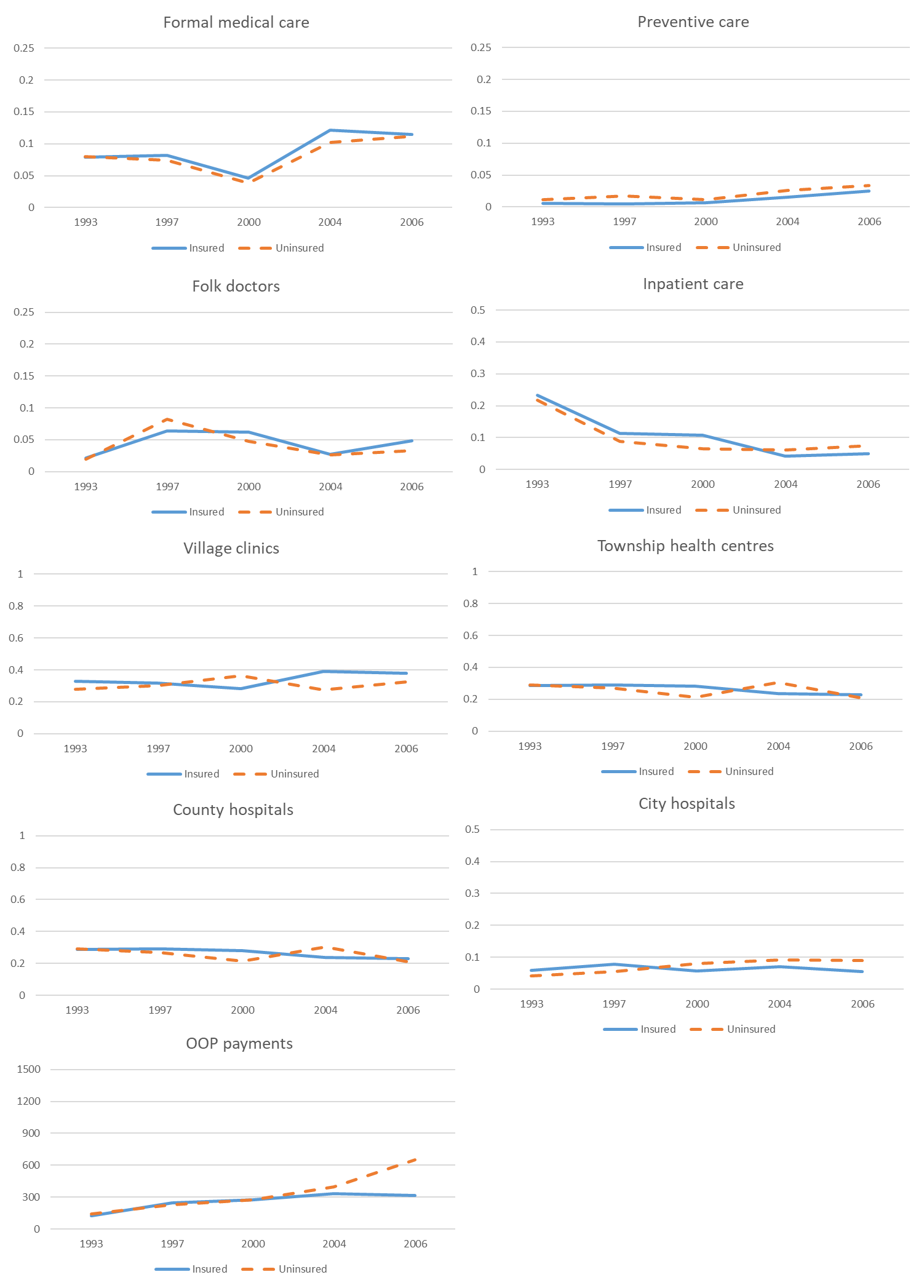
**
